# PIV and PILE Score at Baseline Predict Clinical Outcome of Anti-PD-1/PD-L1 Inhibitor Combined With Chemotherapy in Extensive-Stage Small Cell Lung Cancer Patients

**DOI:** 10.3389/fimmu.2021.724443

**Published:** 2021-10-29

**Authors:** Ran Zeng, Fang Liu, Chen Fang, Jin Yang, Lifeng Luo, Ping Yue, Beili Gao, Yuchao Dong, Yi Xiang

**Affiliations:** ^1^ Department of Respiratory and Critical Care Medicine, Ruijin Hospital, Shanghai Jiao Tong University School of Medicine, Shanghai, China; ^2^ Respiratory and Critical Care Medicine Department, Changhai Hospital, Naval Medical University, Shanghai, China; ^3^ Institute of Respiratory Diseases, Shanghai Jiao Tong University School of Medicine, Shanghai, China

**Keywords:** anti-PD-1/PD-L1 inhibitors, small cell lung cancer, Pan-Immune-Inflammation Value, PILE, biomarker

## Abstract

**Objectives:**

The objective of this study is to evaluate whether PIV (Pan-Immune-Inflammation Value) and PILE [a score derived from PIV, lactate dehydrogenase (LDH), and Eastern Cooperative Oncology Group Performance Status (ECOG PS)] can predict clinical outcome of anti-PD-1/PD-L1 inhibitor combined with chemotherapy in patients with extensive-stage (ES) small cell lung cancer (SCLC).

**Methods:**

A total of 53 patients with ES-SCLC in the control group of clinical trial (NCT03041311) were included in this study. PIV was calculated as follows: (neutrophil count × platelet count × monocyte count)/lymphocyte count. The PILE scores were composited based on PIV, LDH levels, and ECOG PS. The Kaplan–Meier method and Cox hazards regression models were used for survival analyses. Moreover, the predictive ability of PIV and PILE was validated in an independent real-world group consisting of 84 patients.

**Results:**

Patients in the low PIV group (PIV < median) had longer progression-free survival (PFS) and overall survival (OS) than those in the high PIV group (PIV ≥ median), along with the HR, which was 2.157 and 2.359, respectively (PFS HR 95% CI: 1.181–3.940, p = 0.012; OS HR 95% CI: 1.168–4.762, p = 0.020). High PILE score was observed relating to worse treatment efficacy (disease control rate (DCR): 84.21% *vs*. 100%, p = 0.047; durable clinical benefit (DCB) rate: 10% *vs*. 48.5%, p = 0.060) and poor clinical outcome (median PFS: 4.75 *vs*. 5.53 m, p = 0.043; median OS: 7.13 *vs*. 15.93 m, p = 0.002). Similar results were obtained about the predictive and prognostic abilities of PIV and PILE scores in the validation group.

**Conclusions:**

High PIV and high PILE were correlated with worse clinical outcomes in ES-SCLC patients treated with anti-PD-1/PD-L1 inhibitor combined with chemotherapy, reflecting that PIV and PILE might be useful to identify patients unlikely to benefit from anti-PD-1/PD-L1 therapy.

## Introduction

Lung cancer belongs to the category of malignant tumors with the highest morbidity and mortality, and the two major types are non-small cell lung cancer (NSCLC) and small cell lung cancer (SCLC) (1). SCLC is more aggressive and fatal than NSCLC. According to the definition of Veterans Administration Lung Study Group, SCLC is usually classified into two stages: limited and extensive stages (LS and ES) (2). Unfortunately, before the era of immunotherapy, 70% patients diagnosed with SCLC were already in the ES, for whom the standard first-line treatment remained to be platinum-based doublet chemotherapy (3, 4). Despite the initial encouraging benefit of chemotherapy, the 1-year progression rate is 94% among ES-SCLC patients treated with chemotherapy because of chemoresistance (5).

Recently, with the promising efficacy of anti-programmed cell death-1/ligand-1 (PD-1/PD-L1) inhibitor in melanoma, NSCLC, renal cell cancer, and other solid tumors, a variety of clinical trials have been performed to estimate the antitumor performance of immunotherapy in SCLC (6). Checkmate-032, Keynote-028, and Keynote-158 studies reported improved clinical benefit with anti-PD-1 inhibitor (combined or not combined with chemotherapy) as second- or later-line therapy in ES-SCLC (7–9). In the IMpower133, significantly extended overall survival (OS) (median OS: 12.3 *vs*. 10.3 m, HR: 0.76) and progression-free survival (PFS) (median PFS: 5.2 *vs*. 4.3 m, HR: 0.77) were observed in the atezolizumab plus standard chemotherapy group as the first-line treatment in ES-SCLC, as compared with the standard chemotherapy group. Another phase III clinical trial, Caspian study, showed similar results (median OS: 13.0 *vs*. 10.3 m, HR: 0.73) (10–12).

Following chemotherapy, anti-PD-1/PD-L1 inhibitor combined with chemotherapy has become an important first-line treatment strategy for SCLC. However, there is still no ideal predictive factor to identify potential responders to anti-PD-1/PD-L1 treatment in SCLC patients. Several studies showed that PD-L1 tumor proportion score (TPS), tumor mutational burden (TMB), and circulating tumor DNA (ctDNA) might be potential predictors for ES-SCLC. However, the limitations (technique, cost, and sample restriction) strongly restrict practical clinical applications (13–15).

The function of anti-PD-1/PD-L1 inhibitor is enhancing the antitumor ability of cytotoxic T lymphocyte and inhibiting tumor immune escape through blocking the PD-1/PD-L1 pathway. Hence, detecting the systemic immune and cancer-related inflammation status may be better at predicting the response of body and tumor to anti-PD-1/PD-L1 inhibitor. The correlation between systemic immune, cancer-related inflammation status and prognosis has been considered as an established fact (6). A variety of blood and biochemical parameters have been investigated as potential inflammatory biomarkers associated with drug efficacy and prognosis, including neutrophil-to-lymphocyte ratio (NLR), derived NLR (dNLR), platelet-to-lymphocyte ratio (PLR), lymphocyte-to-monocyte ratio (LMR), cytokines, and lactate dehydrogenase (LDH) (13, 16–19).

Pan-Immune-Inflammation Value (PIV), a recently developed immune-inflammation biomarker, was derived from neutrophil count, platelet count, monocyte count, and lymphocyte count. Due to its potential ability to comprehensively reveal systemic immune and cancer-related inflammation status, PIV has been regarded as a reliable predictor of clinical outcomes in advanced cancer patients. Ligorio reported that PIV was an independent predictor of worse OS in patients with HER2-positive advanced breast cancer receiving first-line trastuzumab–pertuzumab biochemotherapy, and PIV outperformed other well-known peripheral blood parameters, such as NLR, PLR, and LMR (20). A pooled analysis also showed that PIV is a new prognostic biomarker in patients with metastatic colorectal cancer treated with first-line therapy and superior to other inflammatory indexes (14, 15).

Furthermore, compound prognostic scores, combining several parameters, have shown promising ability for the prognostic prediction in immunotherapy-receiving patients (21, 22). Clinical and laboratory parameters that are considered as candidate prognostic biomarkers can be concluded to develop a compound prognostic score (23). PILE is a three-parameter score based on the PIV value, Eastern Cooperative Oncology Group Performance Status (ECOG PS), and LDH value. In a study including 120 advanced cancer patients treated with anti-PD-1 or anti-PD-L1 inhibitors for any cancer type, a higher PILE score is associated with decreased PFS and OS, showing PILE as a prognostic score system candidate for immunotherapy (24).

However, there is still no study assessing the efficacy-predictive and prognostic abilities of PIV and PILE at baseline in ES-SCLC patients receiving anti-PD-1/PD-L1 inhibitor combined with chemotherapy. The objective of this study is to evaluate whether baseline PIV and PILE are associated with response to anti-PD-1/PD-L1 inhibitor combined with chemotherapy in ES-SCLC patients.

## Method

### Patient Selection

We intended to include ES-SCLC patients treated with immunotherapy plus chemotherapy as our clinical trial group from Project Data Sphere (PDS), an independent, not-for-profit patient-level-data-sharing platform. Up to June 2021, there were seven clinical trial datasets enrolling SCLC patients shared in PDS, and we included a total of 53 patients with ES-SCLC in the control group of clinical trial (NCT03041311) with complete patient-level records. NCT03041311 was a phase II study of carboplatin, etoposide, and atezolizumab with or without trilaciclib in patients with untreated ES-SCLC. All 53 patients were in the control group and were treated with carboplatin, etoposide, and atezolizumab without trilaciclib.

For the external validation group, we retrospectively collected a total of 317 SCLC patients who were admitted to Ruijin Hospital from January 2015 to February 2021, and 208 SCLC patients who were admitted to Changhai Hospital from January 2018 to February 2021. Patients who met the following criteria were included: 1) pathologically confirmed SCLC; 2) immunotherapy naive; 3) availability of evaluation data; and 4) availability of laboratory data obtained before initiation of anti-PD-1/PD-L1 inhibitor treatment. Finally, a total of 84 SCLC patients treated with anti-PD-1/PD-L1 inhibitor were included, composed of 47 from Ruijin Hospital and 37 from Changhai Hospital.

This study was conducted in accordance with the Declaration of Helsinki, and the protocol was reviewed and approved by the institutional review board of Ruijin Hospital and Changhai Hospital (Approval Number: 2019-72 and B2020-028A).

### Evaluation

For patients from the clinical trial (NCT03041311), the raw individual data about tumor evaluation of the control group were obtained from PDS. For the validation group, chest computed tomography scans were performed every 8–12 weeks according to the administration and additionally as needed to assess disease progression. The time of the last follow-up was in September 2021.

Clinical responses were assessed and categorized as either complete response (CR), partial response (PR), stable disease (SD), or progressive disease (PD), in accordance with the revised Response Evaluation Criteria in Solid Tumors (RECIST) guideline (version 1.1). PFS was defined as the time elapsed between anti-PD-1/PD-L1 inhibitor initiation and tumor progression or death from any cause. OS was defined as the time from the first dose of anti-PD-1/PD-L1 inhibitor to death from any cause. The objective response rate (ORR) and disease control rate (DCR) were defined as CR plus PR, and CR plus PR plus SD, respectively. Duration of response (DOR) was defined as the duration from objective response (CR or PR) to PD in responders. Durable clinical benefit (DCB) was defined as the percentage of patients who achieved CR or PR or SD that lasted >6 months; non-DCB (NDB) was defined as PD or SD that lasted ≤6 months.

### Data Collection

The following data were collected from PDS online and the medical records of Ruijin Hospital and Changhai Hospital: age, sex, ECOG PS, smoking status, brain metastasis status at diagnosis, disease stage at diagnosis, history of treatments, and treatment response. Blood test results before the first administration of anti-PD-1/PD-L1 inhibitor were also recorded. Hematological and biochemistry parameters of interest were as follows: absolute neutrophil count, absolute monocyte count, absolute platelet count, absolute lymphocyte count, and LDH. The PIV was calculated with the following equation [neutrophil count (10^3^/ml) × platelet count (10^3^/ml) × monocyte count (10^3^/ml)]/lymphocyte count (10^3^/ml). The PILE scores were composite score based on PIV, LDH level, and ECOG PS, which was calculated with the sum of individual value (for PIV <median = 0, ≥median = 1; for LDH ≤upper limit of normal (ULN) = 0, >ULN = 1; for ECOG PS <2 = 0, ≥2 = 1).

### Statistical Analysis

The clinical characteristics of included patients were simply expressed as frequencies and percentages for categorical variables. Fisher’s exact test or the chi-square test, as appropriate, was used to analyze the association between baseline PIV and other clinicopathological characteristics. The median follow-up time was estimated using Reverse Kaplan–Meier method. Univariate and multivariate Cox regression analyses were performed to investigate predictive factors for PFS and OS. Factors potentially associated with risk of PFS and OS in the univariate analysis (p ≤ 0.050) were then included for analysis in the multivariate Cox regression analysis. The Kaplan–Meier method was used to assess the cumulative incidence of PFS and OS, and the log-rank test was applied to test for statistical significance. The chi-square test and Fisher’s exact test, as appropriate, were applied to analyze the difference of clinical efficacy (ORR, DCR, DOR, and DCB) between patients with low PILE scores and patients with high PILE scores. A two-tailed p-value <0.05 was considered statistically significant. Statistical analyses were conducted through SPSS 24.0 (IBM, Armonk, NY, USA), GraphPad Prism 8.0 (GraphPad Software, La Jolla, CA, USA), and R, version 3.5.1 (R Foundation for Statistical Computing, Vienna, Austria).

## Results

### Clinical Characteristics According to Pan-Immune-Inflammation Value

The characteristics of the 53 patients from clinical trial (NCT03041311) are outlined in **Table 1**. Median PIV of these patients was 581.95 (IQR, 254.22–987.55). Patients were divided into high and low PIV groups according to the median PIV. Overall, 26 (49.1%) patients had a low PIV, and 27 (50.9%) had a high PIV. As shown in **Table 1**, the clinical characteristics (age, gender, ECOG PS status, brain metastasis status, and smoking status) were similar between high and low PIV groups, except for LDH value (p = 0.013). Compared with patients with high PIV, a lower proportion of patients with low PIV had LDH greater than ULN.

**Table 1:** Comparison of baseline characteristics in the low and high PIV groups.

	Total	Low PIV (<581.95)	High PIV (≥581.95)	p-value
	N (%)	N (%)	N (%)	
Age				
<65 years	27 (50.9)	13 (50)	14 (51.9)	0.893
≥65 years	26 (49.1)	13 (50)	13 (48.1)	
Gender				
Male	34 (64.2)	20 (76.9)	14 (51.9)	0.251
Female	19 (35.8)	6 (23.1)	13 (48.1)	
Brain metastasis at diagnosis				
Yes	14 (26.4)	6 (23.1)	8 (29.6)	0.589
No	39 (73.6)	20 (76.9)	19 (70.4)	
ECOG PS status				
0–1	46 (86.8)	24 (92.3)	22 (81.5)	0.420
2.00	7 (13.2)	2 (7.7)	5 (19.5)	
Smoking status				
Never	6 (11.3)	5 (19.2)	1 (3.7)	0.100
Current or former	47 (88.7)	21 (80.8)	26 (96.3)	
LDH				
≤ULN	25 (47.2)	17 (65.4)	8 (29.6)	**0.013**
>ULN	28 (52.8)	9 (34.6)	19 (70.4)	
Total	53 (100)	26 (49.1)	27 (50.9)	

Bold values indicate statistical significance at the p < 0.050 level.

PIV, Pan-Immune-Inflammation Value; LDH, lactate dehydrogenase; ULN, upper limit of normal; ECOG PS, Eastern Cooperative Oncology Group Performance Status.

### Survival Analysis According to Pan-Immune-Inflammation Value

**Figure 1:**
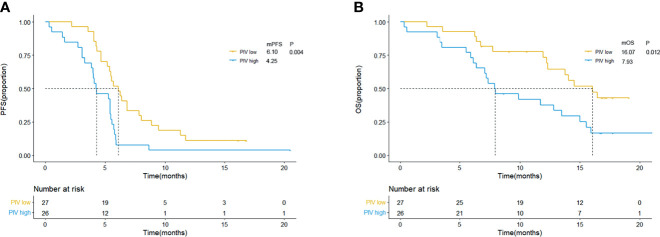
Kaplan–Meier curves for PFS **(A)** and OS **(B)** according to baseline PIV in clinical trial group. Yellow lines indicate patients with low PIV (<581.95), and blue lines indicate patients with high PIV (≥581.95). mOS, median overall survival; mPFS, median progression-free survival; m, month; PIV, Pan-Immune-Inflammation Value.

Patients were separated into high and low PIV groups as mentioned before. As shown in **Figure 1**, compared with patients with low PIV, worse clinical outcome (both PFS and OS) could be observed in patients with high PIV, and the log-rank test showed that the differences were significant [median PFS: 6.10 m (95% CI: 4.86–7.34 m) *vs*. 4.25 m (95% CI: 2.61–5.82 m), p = 0.004; median OS: 16.07 m (95% CI: 12.61–19.77 m) *vs*. 7.93 m (95% CI: 4.60–11.20 m), p = 0.012, **Figure 1**]. And 6-month PFS (51.9% *vs*. 42.3%) and 12-month OS rate (63.0% *vs*. 34.6%) were better in the low PIV group compared with the high PIV group.

Afterwards, the univariate and multivariate analyses are used to evaluate the potential independent predictors, and the results are shown in **Tables 2** and **3**. Factors regarding age, gender, disease stage, ECOG PS, smoking status, brain metastasis status, and hematological and biochemistry parameters of interest (PIV and LDH level) at baseline were included in the univariate analysis. In the univariate analysis, high PIV and higher ECOG PS (≥2) were associated with shorter PFS (PIV: HR = 2.331, 95% CI: 1.296–4.193, p = 0.005; ECOG PS: HR = 2.556, 95% CI: 1.105–5.913, p = 0.028). As for OS, the patients with high PIV had significantly shorter OS than those with low PIV (HR = 2.569, 95% CI: 1.285–5.136, p = 0.015). Patients with high LDH according to ULN had poorer OS than those whose LDH were low or of normal level (HR = 1.974, 95% CI: 1.000–3.896, p = 0.047). Given the limitation of univariate analysis, multivariate analysis was performed to investigate the independent predictive and prognostic factors. Factors that significantly associated with risk of PFS and OS in the univariate analysis were concluded. The result showed that PIV was an independent predictive factor for PFS and OS among the SCLC patients treated with anti-PD-1/PD-L1 inhibitor combined with chemotherapy (PFS: HR = 2.157, 95% CI: 1.181–3.940, p = 0.012; OS: HR = 2.359, 95% CI: 1.168–4.762, p = 0.020).

**Table 2:** Univariate and multivariate analyses of PFS.

Factors	Univariate analysis		Multivariate	
	HR, 95% CI	p-Value	HR, 95% CI	p-Value
Age				
<65	1.00			
≥65	0.995 (0.568–1.745)	0.987		
Gender				
Female	1.00			
Male	0.928 (0.514–1.674)	0.804		
Smoking status				
Never	1.00			
Current or former	2.743 (0.976–7.712)	0.056		
ECOG PS				
0–1	1.00		1.00	
2	2.556 (1.105–5.913)	**0.028**	1.997 (0.853–4.676)	0.111
Brain metastasis at diagnosis				
No	1.00			
Yes	1.382 (0.730–2.615)	0.321		
LDH				
<ULN	1.00			
≥ULN	0.403 (0.721–2.254)	0.403		
PIV				
<581.95	1.00		1.00	
≥581.95	2.331 (1.296–4.193)	**0.005**	2.157 (1.181–3.940)	**0.012**

Bold values indicate statistical significance at the p < 0.05 level.

PIV, Pan-Immune-Inflammation Value; LDH, lactate dehydrogenase; ULN, upper limit of normal; PFS, progression-free survival; ECOG PS, Eastern Cooperative Oncology Group Performance Status.

**Table 3:** Univariate and multivariate analyses of OS.

Factors	Univariate analysis		Multivariate	
	HR, 95% CI	p-Value	HR, 95% CI	p-Value
Age				
<65	1.00			
≥65	1.599 (0.819–3.121)	0.169		
Gender				
Female	1.00			
Male	0.926 (0.465–1.843)	0.827		
Smoking status				
Never	1.00			
Current or former	2.339 (0.560–9.770)	0.244		
ECOG PS				
0–1	1.00			
2	2.281 (0.980–5.311)	0.056		
Brain metastasis at diagnosis				
No	1.00			
Yes	1.382 (0.730–2.615)	0.325		
LDH				
<ULN	1.00		1.00	
≥ULN	1.974 (1.000–3.896)	**0.047**	1.726 (0.866–3.444)	0.106
PIV				
<581.95	1.00		1.00	
≥581.95	2.569 (1.285–5.136)	**0.015**	2.359 (1.168–4.762)	**0.020**

Bold values indicate statistical significance at the p < 0.050 level.

PIV, Pan-Immune-Inflammation Value; LDH, lactate dehydrogenase; ULN, upper limit of normal; OS, overall survival; ECOG PS, Eastern Cooperative Oncology Group Performance Status.

### Predictive and Prognostic Ability Evaluation of PILE

Based on the univariate analysis of 53 patients from clinical trial (NCT03041311), ECOG PS and LDH are associated with PFS and OS, respectively, though neither of them are independent predictive factors. Considering the ECOG PS and LDH were candidate prognostic factors in several cancer types, we further evaluated the predictive and prognostic abilities of PILE, a candidate prognostic score, among anti-PD-1/L1 inhibitor-treated patients with SCLC.

**Figure 2:**
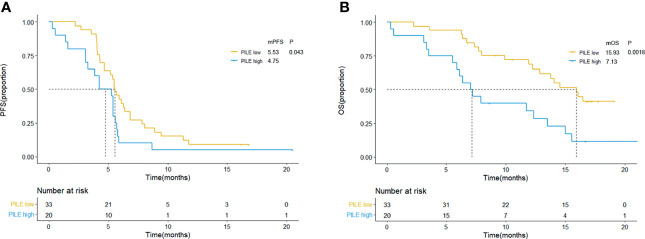
Kaplan–Meier curves for PFS **(A)** and OS **(B)** according to baseline PILE in clinical trial group. Yellow lines indicate patients with low PILE (PLIE score = 0, 1); blue lines indicate patients with high PILE (PLIE score = 2, 3). mOS, median overall survival; mPFS, median progression-free survival; m, month.

The scoring method of PILE is described in the Method section, which used a simple 0/1 scoring system (for PIV < median = 0, ≥median = 1; for LDH ≤ULN = 0, >ULN = 1; for ECOG PS <2 = 0, ≥2 = 1). Thus, the highest score of PILE is 3. Fifty-three patients were categorized into the low PILE group (PILE = 0–1) and high PILE group (PILE = 2–3). A total of 33 (62.3%) patients were in the low PILE group, and 20 (37.7%) patients were in the high PILE group. Significantly shorter PFS and OS were observed in the high PILE group [median PFS (mPFS): 5.53 m (95% CI: 4.93–6.13 m) *vs*. 4.75 m (95% CI: 2.14–6.33 m), p = 0.043; median OS (mOS): 15.93 m (95% CI: 12.95–18.92 m) *vs*. 7.13 m (95% CI: 5.24–8.93 m), p = 0.002, **Figure 2**].

**Figure 3:**
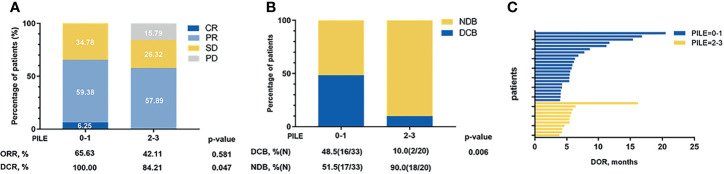
The relations between PILE and clinical efficacy of immunotherapy. **(A)** Overall best response in low and high PILE groups. **(B)** DCB and NDB patients in low and high PILE groups. **(C)** DOR for patients in low PILE group and high PILE groups. CR, complete response; PR, partial response; SD, stable disease; PD, progression disease; ORR, objective response rate; DCR, disease control rate; DOR, duration of response; DCB, durable clinical benefit; NDB, non-durable clinical benefit.

Additionally, the relations between PILE and clinical efficacy were estimated, including ORR, DCR, DOR, and DCB. Among 53 patients, median DOR was 5.57 months, and 18 (34.0%) were evaluated as DCB patients. The ORR and DCR were higher in the low PILE group than the high PILE group (65.63% *vs*. 42.11%; 100% *vs*. 84.21%, **Figure 3A**). As shown in **Figure 3B**, there was a trend towards longer DOR in patients with low PILE score, and a significantly higher proportion of DCB patients were observed in the PILE low group (48.5% *vs*. 10%, p = 0.006, **Figure 3C**).

### Performance of PIV and PILE in Real-World Validation Group

In order to verify the practical predictive and prognostic abilities of PIV and PILE scores among SCLC patients treated with anti-PD-1/L1 inhibitors, we further enrolled 84 SCLC patients treated with anti-PD-1/L1 inhibitors as an independent validation group from Ruijin Hospital and Changhai Hospital. The characteristics of these 84 patients are shown in **Table S1**. The results of Cox regression of PFS and OS are presented in **Table S2**. With a median follow-up time of 14 months (95% CI: 12.942–15.058 m), a total of 65 (77.4%) tumor progression events and 53 (63.1%) death events were observed, with a median PFS of 5.72 months and a median OS of 7.7 months, respectively.

**Figure 4:**
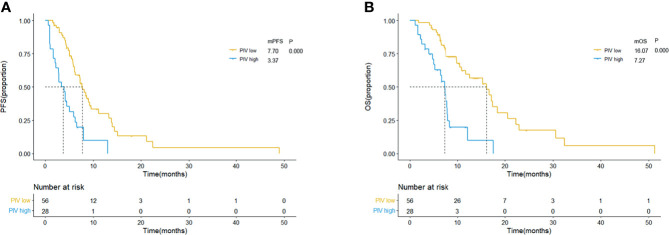
Kaplan–Meier curves for PFS **(A)** and OS **(B)** according to baseline PIV in external validation group. Yellow lines indicate patients with low PIV (<581.95), and blue lines indicate patients with high PIV (≥581.95). mOS, median overall survival; mPFS, median progression-free survival; m month; PIV, Pan-Immune-Inflammation Value.

**Figure 5:**
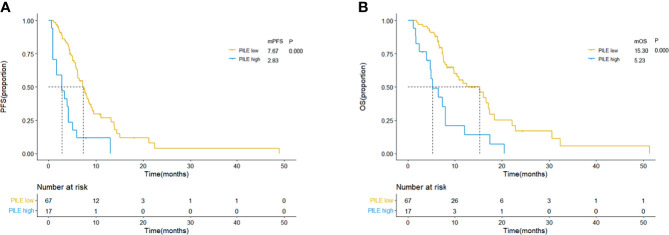
Kaplan–Meier curves for PFS **(A)** and OS **(B)** according to baseline PILE in external validation group. Yellow lines indicate patients with low PILE (PLIE score = 0, 1); blue lines indicate patients with high PILE (PLIE score = 2, 3). mOS, median overall survival; mPFS, median progression-free survival; m, month.

The comparison of therapeutic effect revealed that the high PILE group had lower DCR and DCB rates in real-world validation group (ORR: 5.88% *vs*. 43.94%, p = 0.004; DCR: 94.42% *vs*. 35.92%, p = 0.000, **Figure S1**). According to the Kaplan–Meier methods and log-rank analysis, similar results about the predictive and prognostic abilities of PIV and PILE scores were obtained. SCLC patients with high PIV had a shorter PFS and OS than had those with low PIV (mPFS: 3.37 *vs*. 7.70 m, p = 0.000; mOS: 7.27 *vs*. 16.07 m, p = 0.000; **Figures 4A, B**). High PILE score (PILE = 2, 3) was also correlated with worse clinical outcome (mPFS: 2.83 *vs*. 7.67 m, p = 0.000; mOS: 5.23 *vs*. 15.30 m, p = 0.000; **Figures 5A, B**).

The results of Cox regression analysis also showed that PIV was an independent predictive factor for PFS and OS among the external validation group (PFS: HR = 2.162, 95% CI: 1.182–3.947, p = 0.012; OS: HR = 2.888, 95% CI: 1.509–5.496, p = 0.001, **Tables S2** and **S3**). Additionally, we applied survival analysis to patients from different hospitals for further validation. The results were all consistent to the results above (**Figures S2** and **S3**).

## Discussion

The introduction of anti-PD-1/PD-L1 inhibitor has expanded the treatment options for SCLC patients. Several II and III clinical trials proved the antitumor efficacy of anti-PD-1/PD-L1 combined with chemotherapy. As shown in the results of IMpower133 and Caspian studies, immunotherapy plus chemotherapy significantly increased the 1-year OS rate (IMpower133: 51.7% *vs*. 38.2%; Caspian: 54% *vs*. 50%) (10, 12). However, immunotherapy plus chemotherapy only prolonged OS time by about 2 months, and no significant difference in OS between the two groups in the early phase was noted. Worse than this, the application of anti-PD-1/PD-L1 in SCLC patients is further shadowed by the life-threatening immunotoxicities. Hence, it is of great importance to identify factors to predict the potential responder to anti-PD-1/PD-L1 inhibitor combined with chemotherapy in SCLC patients (25).

PD-L1 TPS and TMB have been widely accepted as efficacy-predictive factors for immunotherapy in several cancers, such as NSCLC and melanoma (16, 17). But both two factors are derived from tumor tissues, which are accompanied by spatial and temporal heterogeneity. Research comparing the PD-L1 TPS of primary lesions to metastatic lesions showed that the difference was significant, and the application of TMB has the same restrictions (13). The result of Checkmate-032 demonstrated that clinical response does not relate to PD-L1 expression, and negative or low PD-L1 expression patients also could benefit from immunotherapy, which indicates that PD-L1 TPS might not be a reliable indicator (8). As a measurement of the number of mutations carried by tumor cells, TMB could be a candidate as a predictive factor of immunotherapy in genomic-instable cancers, including SCLC (18). Studies showed that SCLC patients with higher TMB were more likely to benefit from anti-PD-1 inhibitor (19, 20). Another factor, ctDNA, was strongly associated with the prognosis of SCLC patients treated with second-line immunotherapy, but the technique [next-generation sequencing (NGS)] and expensive cost of TMB and ctDNA evaluations strongly restrict practical clinical applications (13–15).

In the study enrolling SCLC patients with second- or later-line immunotherapy, the median PFS was longer in patients with NLR < 5 than in patients with NLR > 5 at 6 weeks post treatment (16). A study enrolled ES-SCLC patients in a phase II trial, and their results showed that pretreatment PLR could serve as a valuable independent prognostic factor for ED-SCLC patients treated with anti-PD-L1 inhibitor plus chemotherapy (17). Previous studies have reported that dNLR and LDH level were independently associated with PFS, OS in chemotherapy, radiotherapy, and targeted therapy (PARP inhibitors) (26, 27). Other immune- and inflammation-related parameters such as LMR, systemic inflammation response index (SIRI), and lung immune prognostic index (LIPI) have been reported as predictive biomarkers in patients treated with anti-PD-1/L1 inhibitor (17, 28, 29).

Our study evaluated baseline PIV and PILE of ES-SCLC patients treated with anti-PD-1/PD-L1 inhibitor combined with chemotherapy, aiming to find out easy-to-use biomarkers. Our results showed that patients in the low PIV group (PIV < 581.95) had longer PFS and OS than those in the high PIV group (PIV ≥ 581.95), along with HR of 2.157 and 2.359, respectively (95% CI: 1.200–8.742, p = 0.02; 95% CI: 1.200–8.742, p = 0.02). High PILE score was related with worse treatment efficacy (DCB 10% *vs*. 48.5%, p = 0.006) and poor clinical outcome (median PFS: 4.75 *vs*. 5.53 m, p = 0.043; median OS: 7.13 *vs*. 15.93 m, p = 0.002).

Considering routinely hematological parameters reflecting systemic immune and inflammation, PIV is a recently developed biomarker, based on neutrophils, monocytes, platelets, and lymphocytes, and was strongly associated with the clinical outcomes, both PFS and OS, of immunotherapy-treated patients with several types of cancers. In colorectal cancer, breast cancer, renal cancer, melanoma, and NSCLC patients treated with immunotherapy, high PIV was a strong predictor for poorer PFS and OS (14, 15, 20, 21). Compared with separate blood cell parameters, PIV might be able to reveal the complexity of the systemic immune and inflammatory status more comprehensively. The different components of PIV regulate and represent the different aspects of antitumor immunity, which explained the reason why PIV outperformed those established immune- and inflammation-related biomarkers.

Compound score system has been widely performed in advanced cancer patients for prognostic prediction, including immunotherapy (21–23). These comprehensive biomarkers are developed based on clinical and laboratory parameters, the number of which varied between two and seven. ECOG PS and LDH have been widely accepted as prognostic factors, and both are the parameters most frequently concluded in the scoring system (30, 31). PILE is a prognostic candidate score consisting of PIV, LDH, and ECOG PS, which was developed by a simple 0/1 scoring system (for PIV < median = 0, ≥median = 1; for LDH ≤ULN = 0, >ULN = 1; for ECOG PS <2 = 0, ≥2 = 1). A previous study has concluded that PILE was more successful in predicting the clinical outcome for immunotherapy (24). Hence, in our study, we were interested not only in the ability of PIV but also in the performance of PILE, though ECOG PS and LDH were only significantly associated with PFS and OS, respectively, in univariable analysis, which might be due to the small sample size.

As described in the *Results* section, the differences of clinical outcome between groups based on PIV and PILE in real-world validation group seemed to be a little different from the results in the clinical trial group, the reason for which might be that there was, in clinical practice, a tendency to perform immunotherapy in patients with better baseline status. Moreover, different to the clinical trial, the validation group consists of patients with anti-PD-1/L1 inhibitor, first-line, or multi-line. Although Cox regression analysis was performed, it did not show the association between drug agent, treatment history, and clinical outcome; the relation between these factors and treatment efficacy should be further analyzed in large-sample prospective researches.

ECOG PS ≥2 was a strong independent predictor of poor response and survival in patients treated with anti-PD-1 inhibitor (26). Considering that most clinical trials excluded patients with ECOG PS ≥2, the dramatic underrepresentation of patients with ECOG PS ≥2 makes it difficult to ascertain the real benefit of anti-PD-1/L1 inhibitor in this patient population (27). Moreover, the majority of SCLC patients already have ES disease when they were first diagnosed, in which there were more patients of ECOG PS 2–3 (30). Hence, we decided ECOG PS = 2 as a cutoff value, instead of ECOG PS = 1 reported in the previous literature (24).

To our knowledge, this study is the first to evaluate the predictive and prognostic abilities of baseline PIV to immunotherapy combined with chemotherapy in ES-SCLC patients. Moreover, we explored the performance of PILE, a candidate prognostic score based on PIV, LDH, and ECOG PS. Our study showed that PIV and PILE can predict clinical outcome to anti-PD-1/PD-L1 inhibitor combined with chemotherapy in ES-SCLC patients. Furthermore, we validated the performance of PIV and PILE in a real-world patients’ group, and the results are consistent with our previous findings.

Our study has several limitations. First, our study is a retrospective analysis. Although there remains to be a possible selection bias, we have concluded SCLC patients from a clinical trial and two treatment centers to validate the performance of our related factors, PIV and PLIE. In addition, the small sample size might not represent the entire SCLC population, but our external validation group from the real world could have revealed the true status of SCLC patients who received anti-PD-1/L1 inhibitor to some degree. Second, it is significant to notice that the follow-up time for the external group is potentially insufficient. Although the median follow-up time was 14 months, more than half of the death events have been observed in our external validation group, and the predictive and prognostic abilities of PIV and PILE score in SCLC patients receiving immunotherapy plus chemotherapy have been successfully validated. Third, whether the cutoff value of our study is appropriate and what method to identify the optimal value for SCLC patients need to be solved. Therefore, the assessment of PIV and PILE in association with anti-PD-1/PD-L1 inhibitor combined with chemotherapy should be explored in larger sample sizes and future prospective clinical trials.

## Conclusions

PIV, an immune–inflammation biomarker, was correlated with clinical outcomes in ES-SCLC patients treated with anti-PD-1/PD-L1 inhibitor combined with chemotherapy. PILE, a score based on PIV, LDH, and ECOG PS, was a reliable factor for PFS and OS in ES-SCLC patients treated with anti-PD-1/PD-L1 inhibitor combined with chemotherapy, reflecting that PIV and PILE might be useful to identify patients unlikely to benefit from anti-PD-1/PD-L1 therapy plus chemotherapy. Further large sample and prospective studies are necessary to validate our conclusions.

## Data Availability Statement

The original contributions presented in the study are included in the article/**Supplementary Material**. Further inquiries can be directed to the corresponding authors.

## Ethics Statement

The studies involving human participants were reviewed and approved by Ruijin Hospital (Approval Number: 2019-72) and Changhai Hospital (Approval Number: B2020-028A). Written informed consent for participation was not required for this study in accordance with the national legislation and the institutional requirements. Written informed consent was not obtained from the individual(s) for the publication of any potentially identifiable images or data included in this article.

## Author Contributions

YX, BG, and YD conceived and designed the study. RZ, CF, and JY analyzed the data and wrote and revised the paper. RZ and PY prepared the figures and tables. RZ, FL, CF, JY, LL, and PY collected the data. All authors contributed to the article and approved the submitted version.

## Funding

This work was supported by the National Key R&D Program of China [grant number 2018YFC1311902], Shanghai Key Discipline for Respiratory Diseases [2017ZZ02014], and National Natural Science Foundation of China (81672271).

## Conflict of Interest

The authors declare that the research was conducted in the absence of any commercial or financial relationships that could be construed as a potential conflict of interest.

## Publisher’s Note

All claims expressed in this article are solely those of the authors and do not necessarily represent those of their affiliated organizations, or those of the publisher, the editors and the reviewers. Any product that may be evaluated in this article, or claim that may be made by its manufacturer, is not guaranteed or endorsed by the publisher.
