# Supplementary material for: PIV and PILE Score at Baseline Predict Clinical Outcome of Anti-PD-1/PD-L1 Inhibitor Combined With Chemotherapy in Extensive-Stage Small Cell Lung Cancer Patients
Source: Front Immunol. 2021 Oct 29;12:724443. doi: 10.3389/fimmu.2021.724443 (PMC8586214; doi:10.3389/fimmu.2021.724443)
Supplement: Supplementary file 1 [file DataSheet_1.pdf]

Supplementary Material

1 Supplementary Figures and Tables

1.1 Supplementary Figures

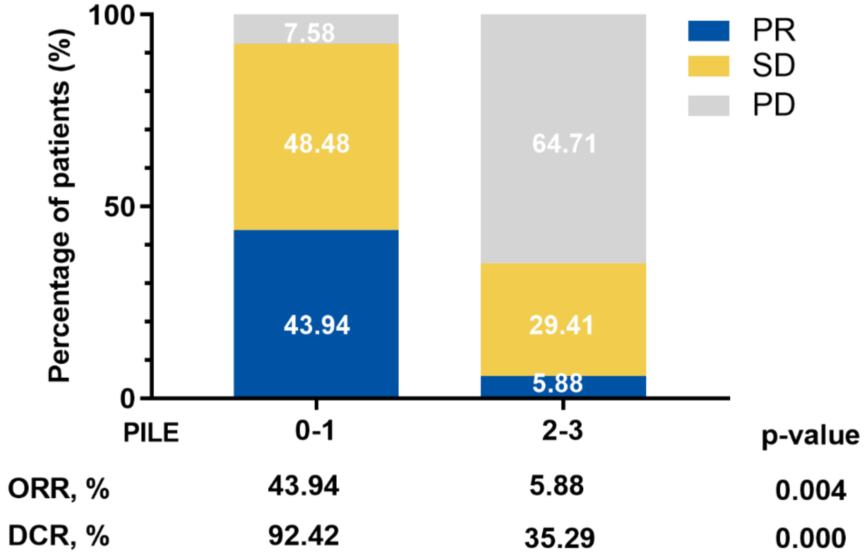

Supplementary Figure 1 The relations between PILE and clinical efficacy of immunotherapy in external validation group

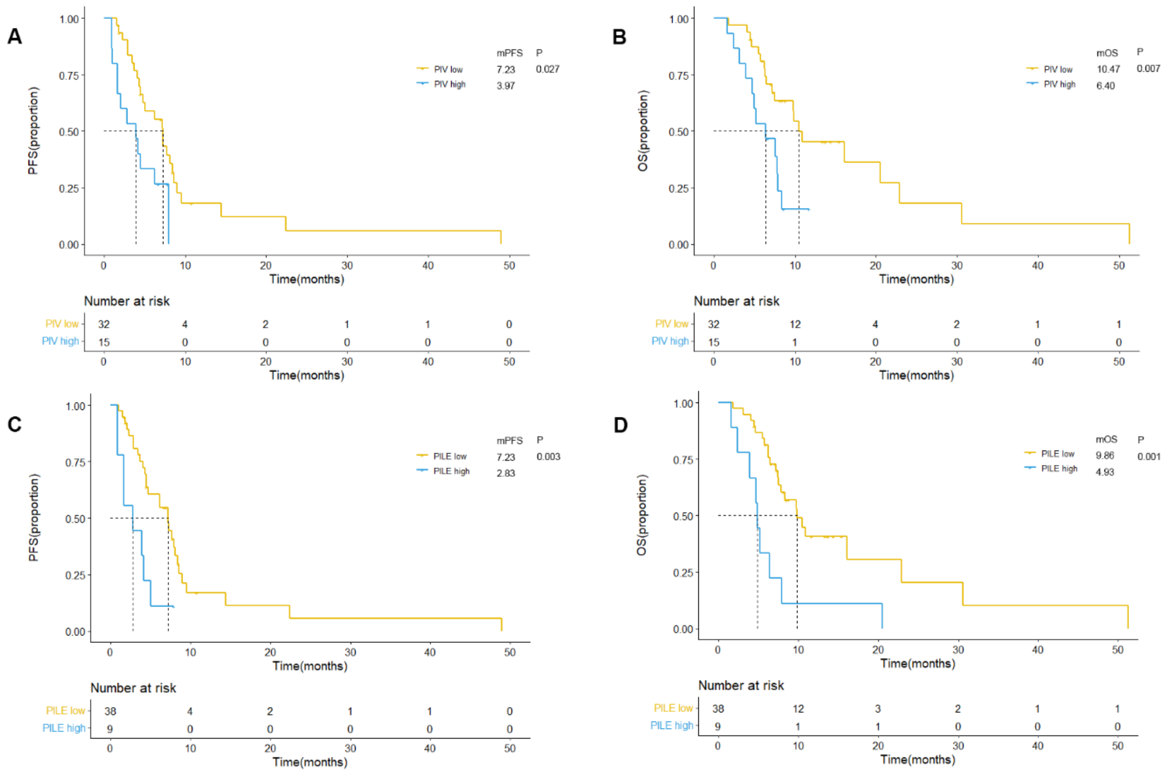

Supplementary Figure 2 Kaplan Meier curves for PFS and OS according to baseline PIV and PILE score in Ruijin Cohort. (A) PFS in low and high PIV group for Ruijin Cohort; (B) OS in low and high PIV group for Ruijin Cohort; (C) PFS in low and high PILE group for Ruijin Cohort; (A) OS in low and high PILE group for Ruijin Cohort.

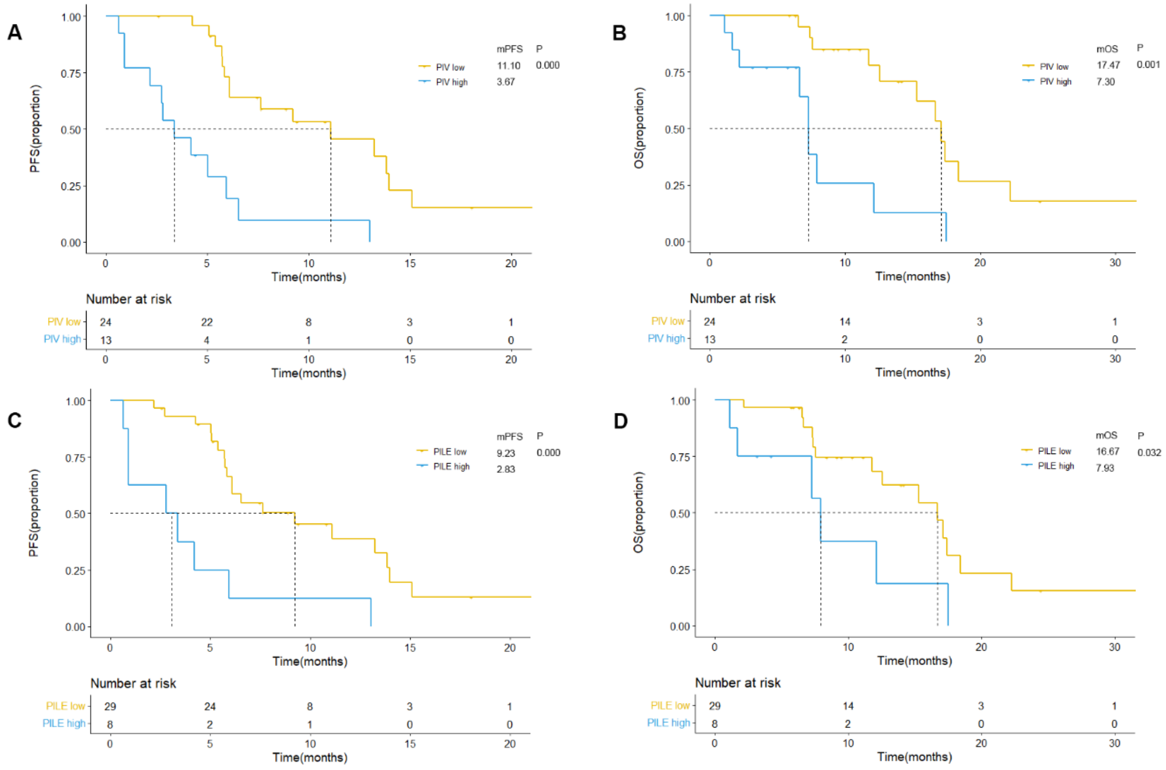

Supplementary Figure 3 Kaplan Meier curves for PFS and OS according to baseline PIV and PILE score in Changhai Cohort. (A) PFS in low and high PIV group for Changhai Cohort; (B) OS in low and high PIV group for Changhai Cohort; (C) PFS in low and high PILE group for Changhai Cohort; (A) OS in low and high PILE group for Changhai Cohort.

1.2 Supplementary Tables

Supplementary Table 1 Comparison of baseline characteristics in the PIV low and high groups of external real-world group

	Total	PIV low （< 581.95）	PIV high （≥581.95）	p-value
	N (%)	N (%)	N (%)	
Cohort				

Ruijin Hospital	47(56%)	32(57.1)	15(53.6)	0.756
Changhai Hospital	37(44%)	24(42.9)	13(46.4)	
Age				
< 65 years	47(56)	33(58.9)	14(50)	0.437
≥65 years	37(44)	23(41.1)	12(50)	
Gender				
Male	75(89.3)	47(83.9)	28(100)	**0.026**
Female	9(10.7)	9(16.1)	0(0)	
Brain metastasis at diagnosis				
Yes	63(75)	45(80.4)	18(64.3)	0.109
No	21(25)	11(19.6)	10(35.7)	
Stage				
limited Stage	7(8.3)	4(7.1)	1(3.6)	0.661
extensive stage	77(91.7)	52(92.9)	27(96.4)	
ECOG PS status				
0-1	76(90.5)	55(98.2)	21(75)	**0.002**
⩾2	8(9.5)	1(1.8)	7(25)	
Line of treatment				
1	37(44)	21(37.5)	16(57.1)	0.087
⩾2	47(56)	35(62.5)	12(42.9)	
Combined with chemotherapy				
Yes	73(86.9)	49(87.5)	25(89.3)	1.000
No	11(13.1)	7(12.5)	3(10.7)	
Agent				
PD-1 antibody	25(29.8)	18(32.1)	7(25.0)	0.500
PD-L1 antibody	59(70.2)	38(67.9)	21(75.0)	
Smoking status				
Never	19(22.6)	18(32.1)	1(3.6)	**0.003**
Current or former	65(77.4)	38(67.9)	27(96.4)	
LDH				
≤ULN	50(59.5)	37(66.1)	13(46.4)	0.084
> ULN	34(40.5)	19(33.9)	15(53.6)	

Total	84	56	28

PIV pan-immune-inflammation value; SCLC small cell lung cancer; LDH lactate dehydrogenase; ULN upper limit of normal; Bold values indicate statistical significance at p < 0.050 level

Supplementary Table 2 Cox analysis of PFS in external real-world group

Factors	univariate analysis		multivariate analysis	
	HR, 95% CI	p value	HR, 95% CI	p value
Cohort				
Ruijin Hospital	1.000			
Changhai Hospital	0.778(0.471-1.286)	0.328		
Age				
< 65	1.000			
≥65	0.797(0.483-1.316)	0.376		
Smoking status				
Never	1.000		1.000	
Current or former	**2.069(1.051-4.074)**	**0.035**	1.495(0.714-3.132)	0.287
ECOG PS				
0-1	1.000		1.000	
2	**5.020(2.276-11.069)**	**0.000**	**2.579(1.070-6.214)**	**0.035**
Stage				
Limited disease	1.000			
Extensive disease	0.668(0.265-1.682)	0.392		
CNS metastasis				
no	1.000		1.000	
yes	**1.779(1.021-3.102)**	**0.042**	1.730(0/975-3.068)	0.061
Line of treatment				
1	1.000			
≥2	0.668(0.402-1.109)	0.119		
combined				
Yes	1.000			
No	1.386(0.642-2.994）	0.406		

Agent				
PD-1 antibody	1.000			
PD-L1 antibody	0.888(0.518-1.523)	0.666		
LDH				
≤ULN	1.000		1.000	
＞ULN	**1.972(1.193-3.261)**	**0.008**	1.391(0.789-2.453)	0.254
PIV				
low(＜581. 95)	1.000		1.000	
high（≥581. 95）	3.101（1. 801-5.341）	**0.000**	**2.160(1.182-3.947)**	**0.012**

PIV pan-immune-inflammation value; LDH lactate dehydrogenase; ULN upper limit of normal;

Bold values indicate statistical significance at $p < 0.05$ level

Supplementary Table 3 Cox analysis of OS in external real-world group

Factors	univariate analysis		multivariate analysis	
	HR, 95% CI	p value	HR, 95% CI	p value
Cohort				
Ruijin Hospital	1.000			
Changhai Hospital	0.699(0.400-1.221)	0.208		
Age				
< 65	1.000			
≥65	1.017(0.581-1.779)	0.953		

Smoking status				
Never	1.000		1.000	
Current or former	2.380(1.063-5.327)	**0.035**	1.586(0.667-3.769)	0.296
ECOG PS				
0-1	1.000		1.000	
2	2.574(1.199-5.527)	**0.015**	1.383(0.578-3.306)	0.466
Stage				
Limited disease	1.000			
Extensive disease	0.515(0.182-1.454)	0.210		
CNS metastasis				
no	1.000			
yes	1.445(0.777-2.687)	0.245		
Line of treatment				
1	1.000			
≥2	0.942(0.525-1.690)	0.840		
combined				
Yes	1.000			
No	1.552(0.694-3.474)	0.285		
Agent				
PD-1 antibody	1.000			
PD-L1 antibody	1.260(0.662-2.396)	0.481		
LDH	1.000		1.000	
≤ULN	1.824(1.045-3.185)	**0.035**	1.219(0.638-2.327)	0.548
＞ULN				
PIV				
low(＜581.95)	1.000		1.000	
high（≥581.95）	3.608(-1.972-6.600)	**0.000**	**2.880(1.509-5.496)**	**0.001**

PIV pan-immune-inflammation value; LDH lactate dehydrogenase; ULN upper limit of normal;

Bold values indicate statistical significance at $p < 0.05$ level
